# Subchronic Exposure to Low-Dose Cannabidiol (CBD) and Δ^9^-Tetrahydrocannabinol (Δ^9^-THC) Nanoemulsions Suggests Low Renal Toxicity: A Preliminary Study

**DOI:** 10.3390/pathophysiology33030065

**Published:** 2026-09-01

**Authors:** Thiago Guedes Pinto, Barbara dos Anjos Rosario, Pedro Everson Alexandre de Aquino, Glauce Socorro de Barros Viana, Nágila Maria Pontes Silva Ricardo, Edilberto Rocha Silveira, Débora Hellen Almeida de Brito, Dávila Zampieri, Milena de Barros Viana, Daniel Araki Ribeiro

**Affiliations:** 1 Department of Biosciences, Institute of Health and Society, Federal University of São Paulo, UNIFESP, Santos 11015-020, SP, Brazil; guedes.pinto@unifesp.br (T.G.P.); barbara.rosario@unifesp.br (B.d.A.R.); mviana@unifesp.br (M.d.B.V.); 2 Department of Physiology and Pharmacology, Federal University of Ceara, UFC, Fortaleza 60020-181, CE, Brazil; pedroeverson.alexandre@gmail.com (P.E.A.d.A.); gbviana@live.com (G.S.d.B.V.); 3 Department of Organic and Inorganic Chemistry, Federal University of Ceara, Fortaleza 60020-181, CE, Brazil; naricard@ufc.br (N.M.P.S.R.); edil@ufc.br (E.R.S.); ak92@hotmail.com (D.H.A.d.B.); davila@dqoi.ufc.br (D.Z.)

**Keywords:** cannabinoids, CBD, Δ^9^-THC, nanoemulsion, kidney, histopathology, immunohistochemistry, Ki-67, caspase-3

## Abstract

**Background/Objectives:** The increasing therapeutic use of cannabinoid-based formulations highlights the need to better characterize their safety profile, particularly in organs involved in xenobiotic elimination, such as the kidneys. This study evaluated the renal effects of cannabidiol (CBD) and Δ^9^-tetrahydrocannabinol (Δ^9^-THC) nanoemulsions following subchronic oral administration in male Wistar rats. **Methods:** Forty animals (*n* = 8/group) were randomly allocated into five groups: vehicle control (Licuri oil + Pluronic^®^ F127), CBD 2.5 mg/kg, CBD 5 mg/kg, CBD+THC 2.5 mg/kg, and CBD+THC 5 mg/kg. Treatments were administered daily by oral gavage for 21 consecutive days. Kidney samples were analyzed by histopathology and immunohistochemistry (Ki-67, GST-P, and cleaved caspase-3). **Results:** High-dose groups (5 mg/kg) exhibited marked histopathological alterations, including tubular and glomerular damage, hemorrhagic areas, inflammatory infiltration, and necrosis. These groups also showed increased Ki-67 and cleaved caspase-3 immunoexpression compared to controls. GST-P expression was significantly increased only in the CBD 5 mg/kg group. No significant changes were observed in the low-dose groups. **Conclusions:** These findings suggest dose-dependent morphological and molecular alterations in renal tissue associated with cannabinoid nanoemulsion exposure under experimental conditions.

## 1. Introduction

Certain plants capable of altering states of consciousness have played significant roles in rituals and religious practices worldwide throughout history. One such plant is *Cannabis sativa* L., one of the earliest species cultivated in East Asia [1,2]. Taxonomically, *Cannabis sativa* L. is considered a single species with different subspecies, including *C. sativa*, *C. indica*, and *C. ruderalis* [3]. Despite being classified as an illicit drug in some countries, cannabis is widely used by both young people and adults, who report effects such as relaxation, stress reduction, and anxiety relief [4,5].

The widespread use of cannabis is evident across different regions of the world [6]. More than 80 million Europeans aged 15–64 have used cannabis at least once, while nearly 100 million individuals aged 12 years or older in the United States have reported lifetime use [7]. In Brazil, the II National Survey on Alcohol and Drugs (LENAD) reported a lifetime prevalence of approximately 7% among adults and 4% among adolescents [8].

The cannabis plant contains more than 400 biologically active chemicals including complex mixtures of phytochemicals, such as cannabinoids, flavonoids, terpenes, phenols, and alkaloids, whose composition varies according to cultivar, growing conditions and location in the cannabis plant [9,10,11,12]. Such variability may influence the biological and pharmacological effects of cannabis-derived products. Therefore, the medical effects of the plant extracts vary between cultivars and plant material, as well as the entourage effect of cannabis [13]. The most abundant phytocannabinoids are Δ^9^-tetrahydrocannabinol (Δ^9^-THC) and cannabidiol (CBD). Δ^9^-THC is primarily responsible for the psychoactive effects of cannabis, whereas CBD may counteract some of these effects and has been widely investigated for potential therapeutic applications [14,15].

Although the predominant use of cannabis remains recreational, regulated cannabinoid-based formulations have been used to alleviate symptoms associated with various conditions, including chronic pain and neurological disorders [16]. In this context, it is essential to better understand the metabolism and potential toxicity of cannabinoids, particularly in organs involved in xenobiotic elimination. The kidney plays a critical role in this process, as it is responsible for eliminating a substantial proportion of cannabinoid metabolites, making it a potential target for drug-induced toxicity [17,18].

Nanoemulsion-based delivery systems have emerged as an important technological platform for improving the solubility, stability, and bioavailability of highly lipophilic compounds such as cannabinoids. However, these systems may also influence biodistribution and toxicity profiles, highlighting the need for specific toxicological evaluation. Recent studies have demonstrated that nanoemulsion-based cannabinoid formulations significantly improve oral bioavailability and systemic exposure in experimental models [19,20]. Furthermore, in vivo studies have reported dose-dependent pharmacokinetic behavior and metabolic interactions of cannabidiol delivered in nanoemulsion systems [21]. Experimental toxicological evidence also suggests potential tissue-specific effects of cannabinoid nanoemulsions in rodents [22]. Despite these advances, data regarding organ-specific toxicological outcomes, particularly in renal tissue following sub-chronic exposure, remain limited, highlighting the need for further investigations.

It is important to note that most experimental studies, including the present one, evaluate isolated cannabinoids such as CBD rather than full-spectrum cannabis extracts. Whole-plant preparations contain multiple bioactive compounds that may interact synergistically (entourage effect), potentially resulting in different biological responses compared to isolate compounds.

In light of the increasing global consumption of cannabis and the limited data regarding cannabinoid-induced renal effects, this study aimed to evaluate whether exposure to nanoemulsions containing CBD and Δ^9^-THC at different concentrations induces histopathological and immunohistochemical alterations in the kidneys of male Wistar rats.

We hypothesize that, due to their enhanced bioavailability, nanoemulsion-delivered cannabinoids may induce dose-related renal alterations detectable by histopathological and molecular markers under the experimental conditions tested.

## 2. Material and Methods

### 2.1. Experimental Design and Treatment Groups

This study used forty male Wistar rats (8–9 weeks old, weighing approximately 280–300 g; *n* = 8 per group) obtained from the Central Animal Facility (CEDEME) of the Federal University of São Paulo (UNIFESP). The study was approved by the Animal Ethics Committee of the Federal University of São Paulo (UNIFESP) under protocol number 1480150923, which is the same institutional approval used for related experimental procedures previously conducted by our research group.

The animals were housed in polypropylene cages (32 cm × 40 cm) under controlled environmental conditions, including a 12-h light–dark cycle and a temperature of 22 ± 1 °C. A maximum of four animals per cage was maintained. Food and water were provided ad libitum, cages were cleaned every two days, and animals were acclimatized for one week prior to the beginning of the experiment.

Animals were randomly allocated into five experimental groups (*n* = 8/group), defined as follows: control group (CTRL, receiving a vehicle nanoemulsion composed ofLicuri oil, (Caldeirão Grande, Brazil), and Pluronic^®^ F127 (Sigma-Aldrich, USA), without cannabinoids; experimental group 1 (CBD 2.5), treated with cannabidiol (CBD) nanoemulsion at 2.5 mg/kg; experimental group 2 (CBD5), treated with CBD nanoemulsion at 5 mg/kg; experimental group 3 (CBD+THC 2.5), treated with a combined cannabidiol and Δ^9^-tetrahydrocannabinol (Δ^9^-THC) nanoemulsion at 2.5 mg/kg; and experimental group 4 (CBD+THC 5), treated with the same combination at 5 mg/kg. A THC-only group was not included, because the study focused on clinically relevant CBD-based and combined formulations commonly investigated for translational therapeutic purposes.

The selected doses (2.5 and 5 mg/kg) were based on previous experimental studies using cannabinoid nanoemulsions, including work conducted by our research group [18], and were considered exploratory for the evaluation of dose-related effects. Furthermore, the regimen consisted of daily oral gavage for 21 consecutive days. The nanoemulsion preparation was described in a previous study conducted by our research group [18]. Briefly, cannabinoids (THC and CBD) were dissolved in Licuri oil, which served as the oily phase. The aqueous phase consisted of Pluronic^®^ F127, a non-ionic surfactant, dissolved under magnetic stirring. The oil phase was slowly added to the aqueous phase under continuous agitation to obtain a pre-emulsion. Final droplet size reduction was achieved by probe sonication for 3 min (10 s on/10 s off, 70% amplitude), using an ice bath to avoid temperature increase.

The nanoemulsion presented nanometric droplet size, low polydispersity index (PDI), and adequate zeta potential, indicating physicochemical stability, as previously reported. These parameters are relevant as they may influence biodistribution and biological effects. It is important to emphasize that the control group received the vehicle nanoemulsion without cannabinoids, composed of Licuri oil and Pluronic^®^ F127. Therefore, the observed effects are associated with the presence of CBD and/or Δ^9^-THC rather than the formulation matrix itself. The cannabidiol (CBD) and Δ^9^-tetrahydrocannabinol (Δ^9^-THC) used in this study were produced by the Brazilian Association of Medicinal Cannabis (ABRACAM, Brazil) in partnership with the Federal University of Ceará, as previously described [23]. The chemical characterization, purity, and preparation details of the cannabinoid nanoemulsion are provided in the referenced study conducted by our research group [22].

At the end of the experimental period, all animals were deeply anesthetized with a mixture of ketamine (75 mg/kg, Syntec, Brazil), xylazine (10 mg/kg, Syntec Brazil), fentanyl (0.5 mg/kg, Syntec, Brazil), and acepromazine (1 mg/kg, Syntec, Brazil) and euthanized by perfusion. This protocol ensured deep anesthesia and absence of pain, in accordance with ethical guidelines. Subsequently, the right kidney was removed and fixed in 10% buffered formalin for 24 h.

### 2.2. Histopathological Analysis

For the histopathological analysis of the kidney, tissue samples were fixed in 10% buffered formalin (Synth, Brazil) for 24 h. Subsequently, tissues were dehydrated in graded alcohol (Synth, Brazil), cleared in xylene (Synth, Brazil), and embedded in paraffin blocks. Sections of 3 µm thickness were obtained, deparaffinized in xylol, rehydrated in ethanol, and stained with hematoxylin–eosin (H&E).

The evaluation was based on previously described criteria [22], considering lesion severity in the renal cortex using a semi-quantitative scoring system. In this sense, lesions were classified as follows: (0) absence of lesions; (1) mild lesions affecting less than 25% of the tissue; (2) moderate lesions affecting 25–50%; and (3) severe lesions affecting more than 50% of the evaluated area.

For each animal, at least 10 random microscopic fields were analyzed. Histopathological evaluation was performed in a blinded manner by a trained examiner. The parameters evaluated included tissue coagulation necrosis, tubular alterations, glomerular damage, hemorrhagic areas, and inflammatory infiltration.

### 2.3. Immunohistochemical Analysis

The protocol used by Moretti et al. [24] was selected for the following analysis: antigen Ki-67 or MIB-1 (Mindbomb E3 Ubiquitin Protein Ligase 1) (Biocare Medical, Pacheco, CA, USA—1:150), placental glutathione S-transferase (GST-P) (Santa Cruz, San Diego, CA, USA—1:5.000) and cleaved caspase-3 (sc-271759, LOT no. #E2016—Santa Cruz Biotechnology, Dallas, TX, USA). Histological sections of 3 μm thickness were initially subjected to deparaffinization in xylene, followed by rehydration through graded ethanol solutions (99.5%). Antigen retrieval was then performed by treating the slides with a citrate buffer solution (10 nM, pH 6; composed of 0.1 M citric acid and 0.1 M sodium citrate, Synth^®^, São Paulo, Brazil), which was heated in a microwave oven for three cycles of 5 min each.

To block endogenous peroxidase activity, the sections were incubated with 3% hydrogen peroxide (H_2_O_2_). Non-specific binding was subsequently minimized by applying Protein Block (Starr Trek Universal HRP Detection, Biocare Medical^®^) for 30 min, aiming to reduce unspecific protein interactions.

Negative controls were included by substituting the primary antibody with buffer solution during the procedure. After blocking, the slides were rinsed thoroughly with distilled water and incubated with the respective primary antibodies.

Visualization of antigen–antibody complexes was achieved using a 0.05% solution of 3,3′-diaminobenzidine (DAB) chromogen (DAKO^®^ North America Inc., Carpinteria, CA, USA). Counterstaining was finally carried out using hematoxylin for caspase-3 and GST-P, while Fast Green was employed for Ki-67. Immunohistochemical evaluation was performed according to the specific characteristics of each antibody.

For Ki-67 and GST-P, immunopositive cells were quantified as percentage of positively stained cells within randomly selected microscopic fields. For each animal, at least 10 randomly chosen fields from well-preserved cortical regions were analyzed, and results were expressed as mean percentage of positive cells. Cleaved caspase-3 expression was evaluated using a semi-quantitative scoring system (0–3), where 0 = no staining; 1 = mild staining (<10% positive cells); 2 = moderate staining (10–50%); and 3 = strong staining (>50%). All analyses were performed in a blinded manner. Only histological sections with adequate preservation and staining quality were included in the analysis (due to histological quality criteria).

### 2.4. Statistical Methods

Data distribution was assessed using the Shapiro–Wilk test, and homogeneity of variances was evaluated using Levene’s test. Based on data distribution and variable type, all datasets were analyzed using the Kruskal–Wallis test followed by Dunn’s post hoc test. Statistical significance was set at *p* < 0.05. All analyses were performed using GraphPad Prism™ version 6.0.

## 3. Results

### 3.1. Histopathological Findings

Histopathological analysis of renal tissue was carried out in animals treated with varying doses of cannabis oil nanoemulsions containing CBD alone or in combination with THC (CBD+THC). The severity of histopathological alterations was categorized using a semi-quantitative scoring system, as shown in Table 1. The control group (vehicle nanoemulsion without cannabinoids) presented a well-preserved renal structure, with all animals classified as score 0.

Statistically significant differences (*p* < 0.05) were observed between the control group and both groups treated with the higher dose (5 mg/kg), including CBD 5 mg/kg and CBD+THC 5 mg/kg.

In the CBD 5 mg/kg group, all animals were classified as score 3, whereas in the CBD+THC 5 mg/kg group, seven animals received score 3 and one animal received score 2.

Additionally, significant differences were observed between high-dose groups (5 mg/kg) and their respective lower-dose counterparts (2.5 mg/kg).

No comparison with the control group revealed statistically significant differences in either the CBD 2.5 mg/kg or the CBD+THC 2.5 mg/kg groups. In these groups, animals were classified as score 0 or 1.

The main histopathological alterations observed at higher doses included hemorrhagic areas, tubular and glomerular alterations, inflammatory infiltrate, and regions of coagulative necrosis, with occasional glomerular atrophy.

**Figure 1:**
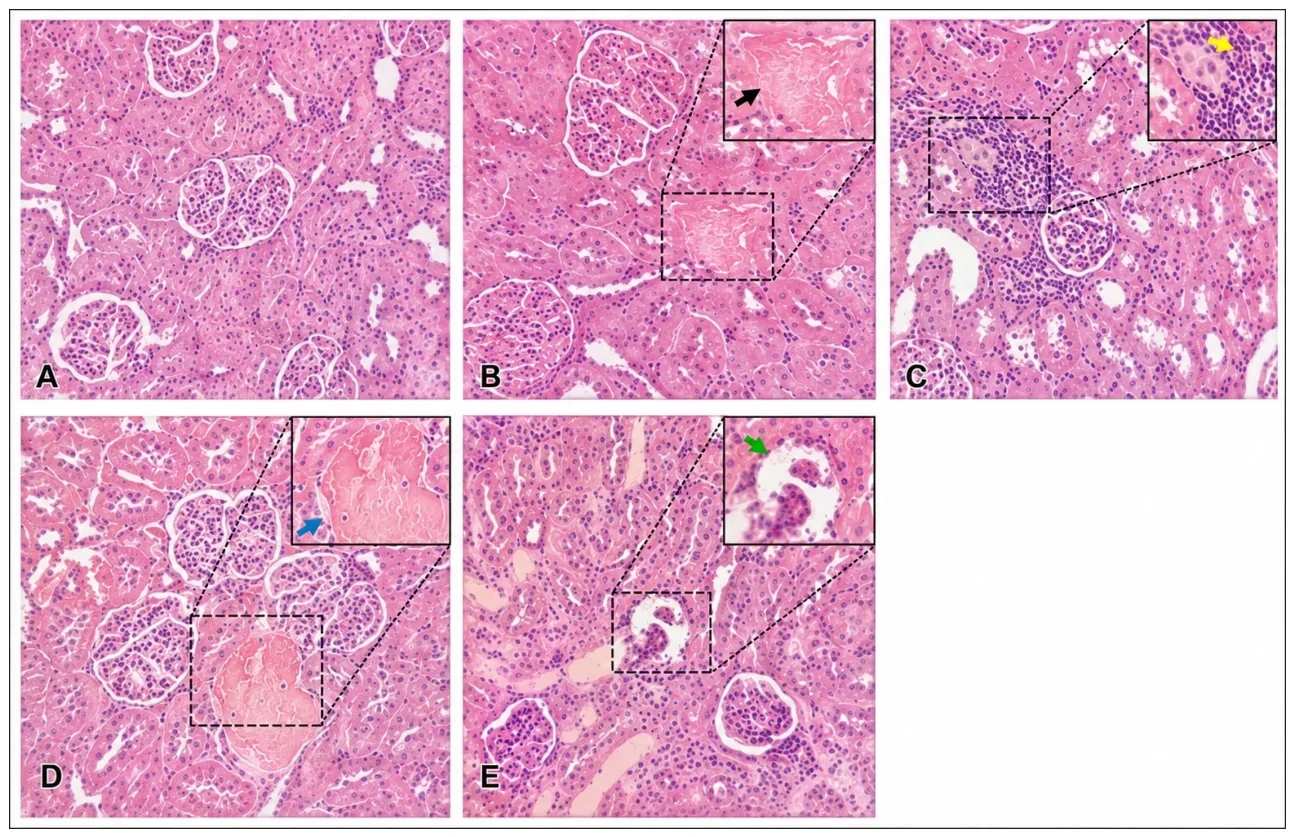
Histopathological analysis of rat kidney tissue following exposure to Cannabis nanoemulsions, demonstrating alterations across all experimental groups included in the study. (**A**) Control group; (**B**) CBD 2.5 mg nanoemulsion group; (**C**) CBD+THC 2.5 mg nanoemulsion group; (**D**) CBD 5 mg nanoemulsion group; (**E**) CBD+THC 5 mg nanoemulsion group. Black arrows denote hemorrhagic areas, yellow arrows indicate inflammatory infiltrate, blue arrows point to glomerular necrosis, and green arrows highlight cellular injury features (hematoxylin and eosin staining, 40× magnification).

These findings are summarized in Table 1 and illustrated in Figure 1.

### 3.2. Immunohistochemical Analysis

Immunohistochemical evaluation of Ki-67 demonstrated a statistically significant increase (*p* < 0.05) in the number of positively stained cells in both groups receiving nanoemulsions at 5 mg/kg (CBD 5 mg/kg and CBD+THC 5 mg/kg) when compared with the control group. This increased Ki-67 immunoexpression was observed in renal tissue of animals exposed to higher cannabinoid doses.

**Figure 2:**
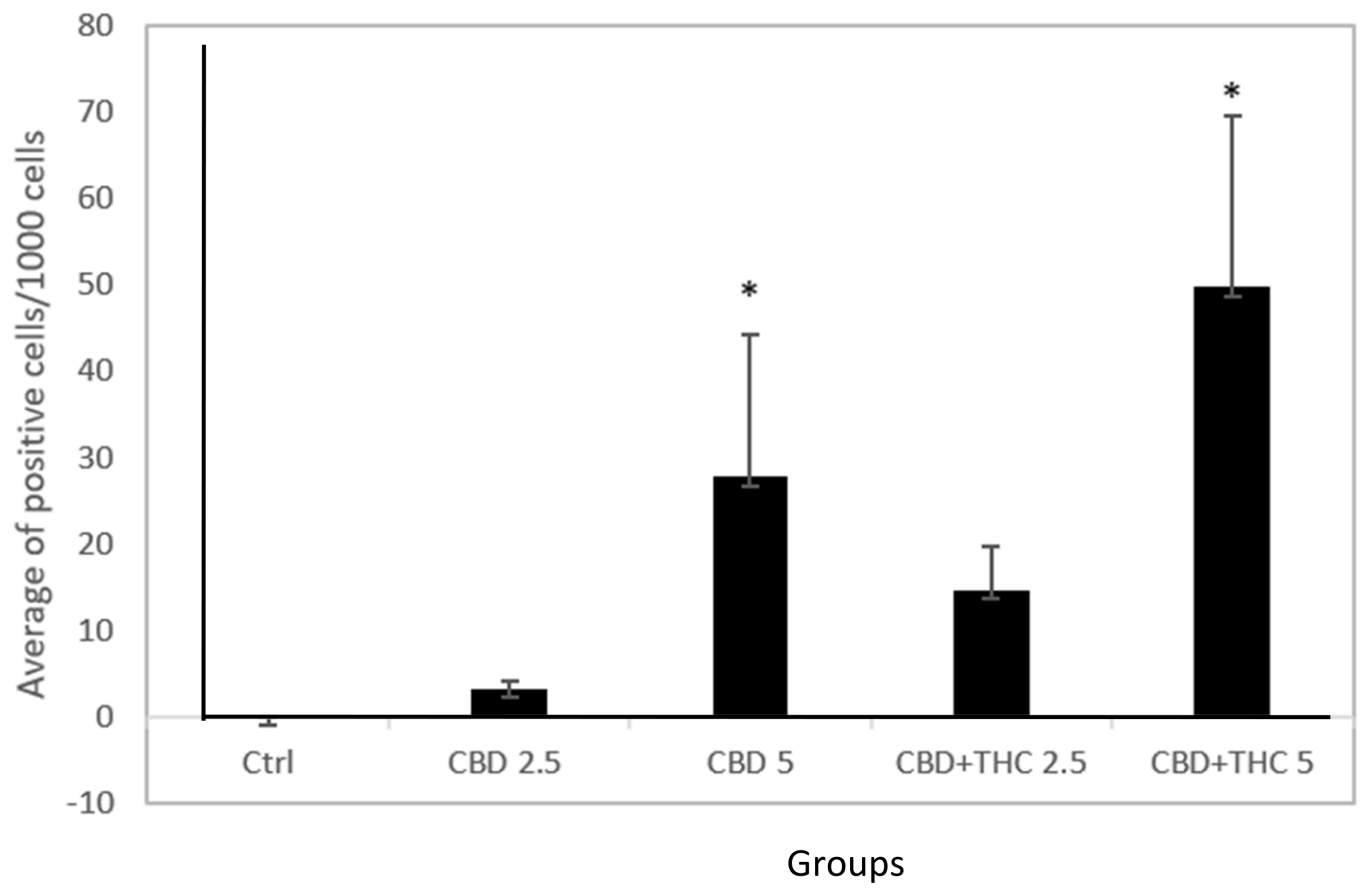
Average number of Ki-67positive cells in rat kidneys following 21 days of treatment with Cannabis oil. Statistically significant differences compared with the control group (*p* < 0.05) are marked with an asterisk (*). Statistical analysis was performed using the Kruskal–Wallis test followed by Dunn’s post hoc test for multiple comparisons.

**Figure 3:**
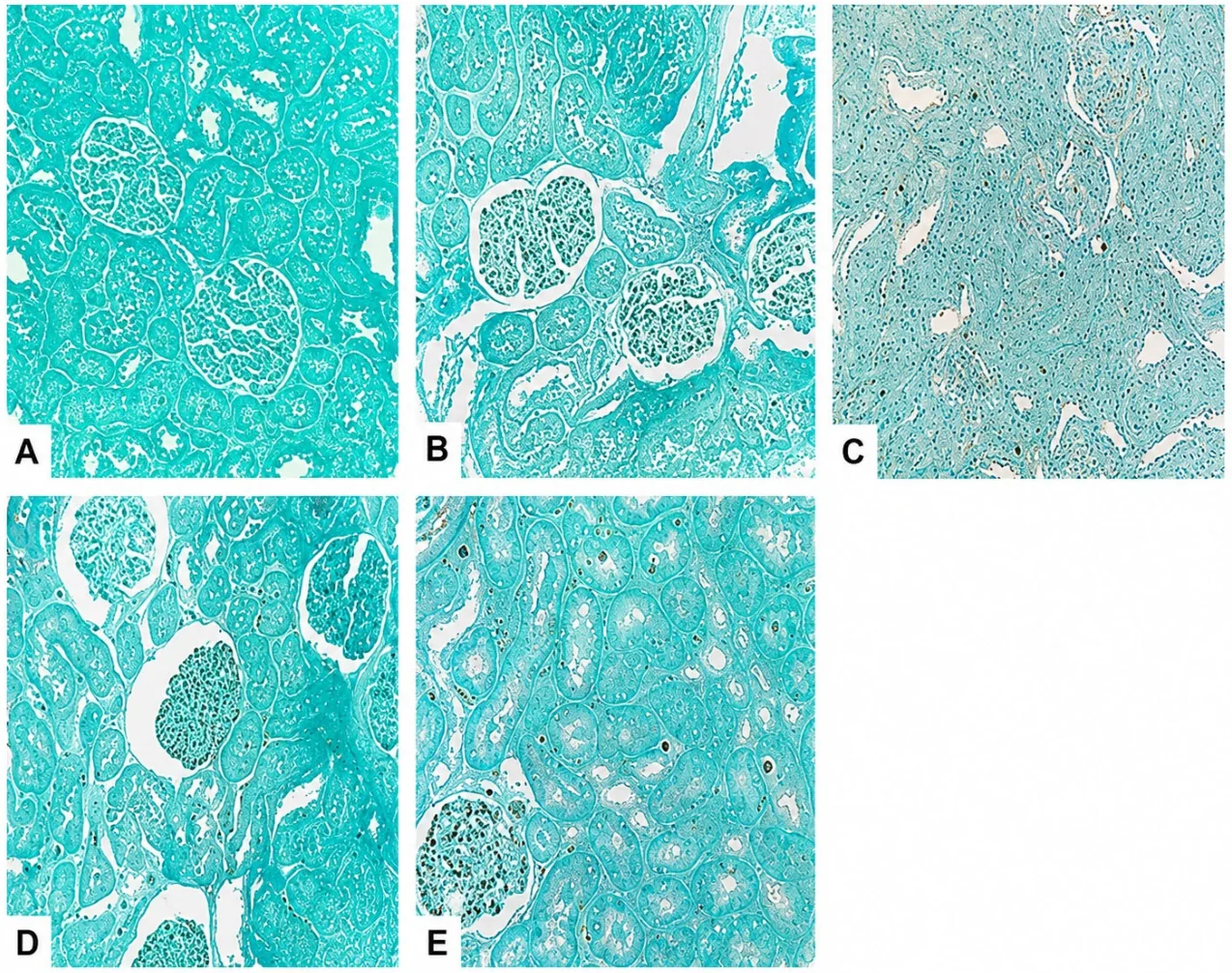
Immunohistochemical detection of Ki-67-positive cells (**A**–**E**) in kidneys of Wistar rats treated with Cannabis nanoemulsions. (**A**) Control group; (**B**) CBD 2.5 mg/kg; (**C**) CBD 5 mg/kg; (**D**) CBD+THC 2.5 mg/kg; (**E**) CBD+THC 5 mg/kg. ABC method, 40× magnification.

In contrast, no statistically significant differences were detected between the control group and the groups treated with lower doses (CBD 2.5 mg/kg and CBD+THC 2.5 mg/kg). Thus, while higher-dose treatments resulted in an increase in Ki-67-positive cells, lower-dose exposure did not produce significant changes. These results are illustrated in Figure 2 and Figure 3.

**Figure 4:**
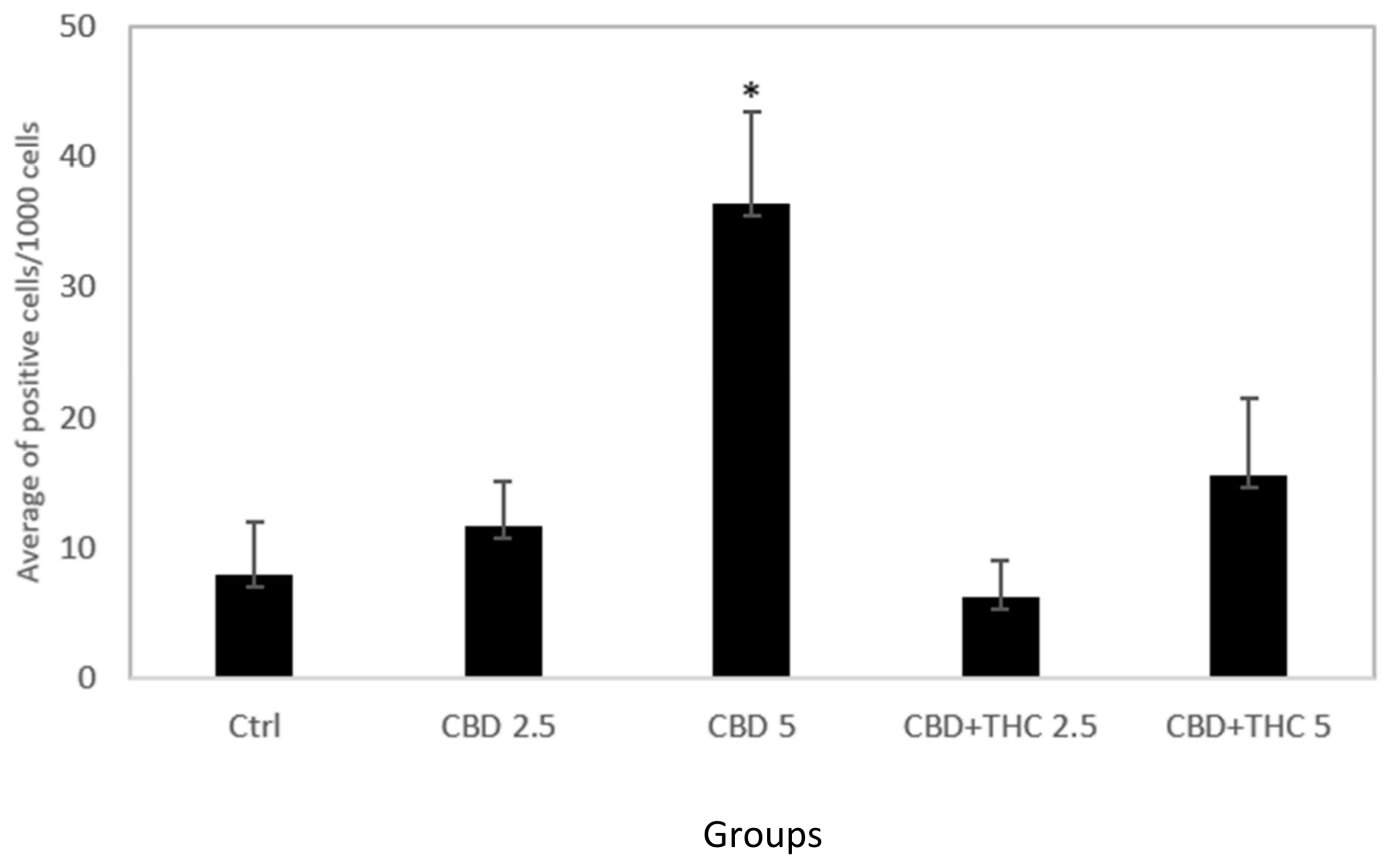
Mean number of GST-P-positive cells in rat kidneys after 21 days of treatment with Cannabis nanoemulsions. Statistically significant increases compared with the control group (*p* < 0.05) are indicated by an asterisk (*). Statistical analysis was performed using the Kruskal–Wallis test followed by Dunn’s post hoc test.

**Figure 5:**
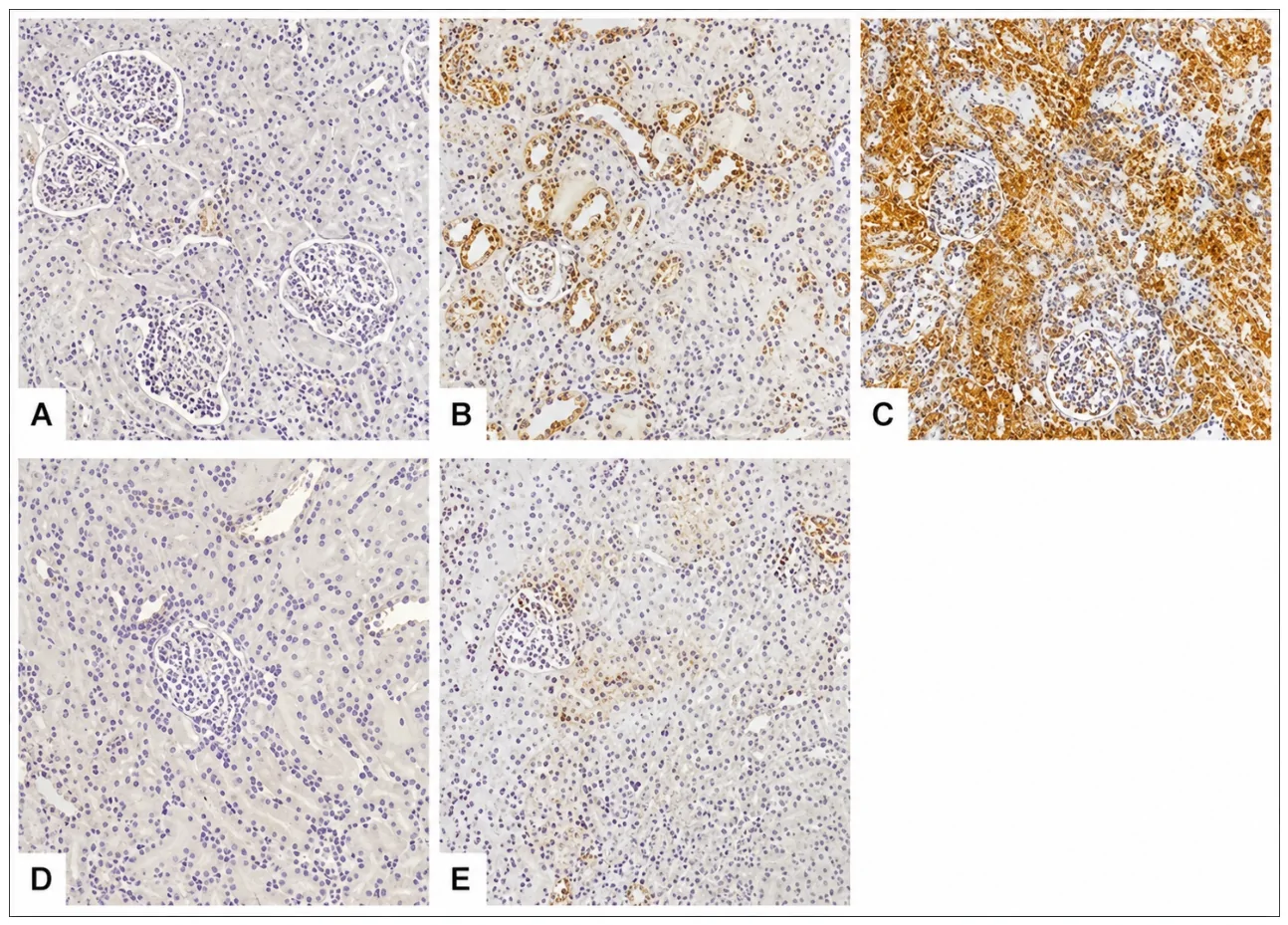
Immunohistochemical analysis of GST-P-positive cells (**A**–**E**) in kidneys of Wistar rats treated with Cannabis nanoemulsions. (**A**) Control group; (**B**) CBD 2.5 mg/kg; (**C**) CBD 5 mg/kg; (**D**) CBD+THC 2.5 mg/kg; (**E**) CBD+THC 5 mg/kg. ABC method, 40× magnification.

Regarding GST-P immunostaining, a statistically significant difference (*p* < 0.05) was observed in the number of positive cells in the group treated with CBD nanoemulsion at 5 mg/kg when compared to the control group. No significant differences were detected for the remaining treatments (CBD 2.5 mg/kg, CBD+THC 2.5 mg/kg, and CBD+THC 5 mg/kg) relative to controls. These results are presented in Figure 4 and Figure 5.

**Figure 6:**
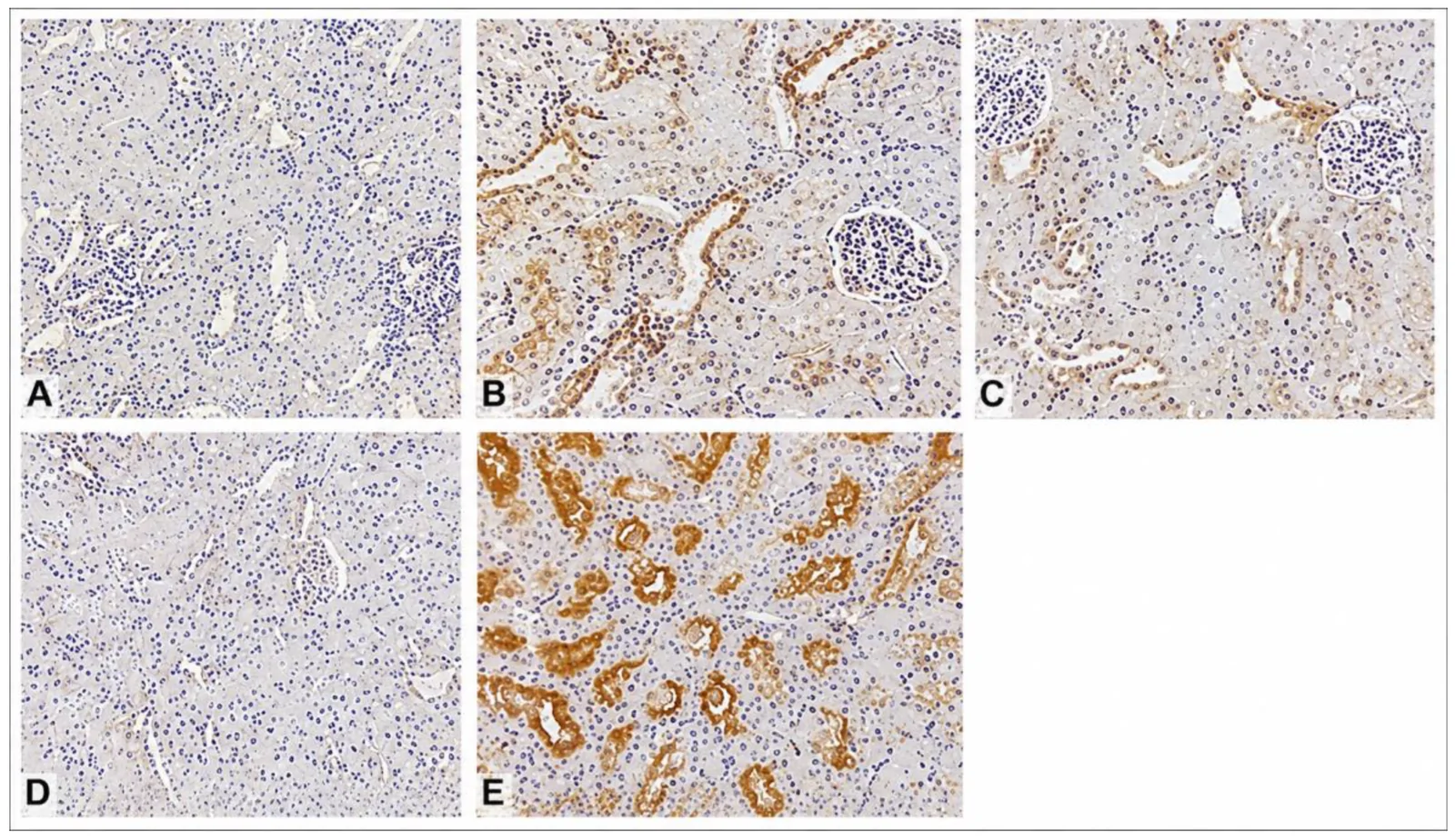
Immunohistochemical detection of cleaved caspase-3-positive cells (**A**–**E**) in kidneys of Wistar rats treated with Cannabis nanoemulsions. (**A**) Control group; (**B**) CBD 2.5 mg/kg; (**C**) CBD 5 mg/kg; (**D**) CBD+THC 2.5 mg/kg; (**E**) CBD+THC 5 mg/kg. ABC method, 40× magnification.

In the cleaved caspase 3 immunohistochemical analysis, we observed statistical differences regarding the amount of immunostained cells in both the CBD only and CBD+THC groups treated with the nanoemulsion at a dose of 5 mg in comparison with the control group (*p* < 0.05), while the lower doses (CBD 2.5 and CBD+THC 2.5 mg/kg) presented no statistical differences when compared with the control group. These findings can be seen in Table 2 and Figure 6.

In the control group, 1 animal received score 0 and 4 received score 1, totaling 5 animals with either score 0 or 1. The same rationale was employed for the other groups. Groups showing statistically significant increases in mean values are marked with an asterisk (*), indicating differences in relation to the control group (*p* < 0.05). Statistical analysis was performed using the Kruskal–Wallis non-parametric test, followed by Dunn’s post hoc test for multiple comparisons.

## 4. Discussion

Given the increasing use of CBD-based formulations and the limited available data regarding their renal safety profile, the present study investigated histopathological and immunohistochemical changes in the kidneys of male Wistar rats following subchronic exposure to cannabinoid-loaded nanoemulsions at different doses.

The histopathological evaluation revealed statistically significant differences between the control group and the groups treated with the higher doses (CBD 5 mg/kg and CBD+THC 5 mg/kg). These alterations were characterized by vascular congestion, hemorrhagic areas, tubular changes, inflammatory infiltrates, and focal structural damage in renal tissue. In contrast, no significant histological changes were observed in the low-dose groups. Importantly, animals receiving the vehicle nanoemulsion alone did not exhibit relevant renal alterations, supporting the interpretation that the observed changes are associated with cannabinoid exposure under the experimental conditions tested.

Similar renal histopathological changes have been previously reported in experimental models exposed to cannabis-derived compounds, including tubular degeneration, vascular alterations, and cellular injury [25]. These findings are consistent with the alterations observed in the present study, particularly at higher doses, suggesting that cannabinoid exposure may be associated with structural changes in renal tissue in a dose-dependent manner. However, it is important to emphasize that histological alterations alone do not necessarily indicate functional renal impairment.

Regarding immunohistochemical findings, increased expression of Ki-67 was observed in the high-dose groups, suggesting increased cellular turnover or regenerative activity in response to tissue alterations. However, Ki-67 is a marker of cell cycle activity and does not distinguish between physiological regeneration and pathological proliferation [26]. Therefore, these findings should be interpreted as indicative of altered cellular dynamics rather than evidence of pathological proliferation per se.

Cleaved caspase-3 immunoexpression was also increased in the high-dose groups, indicating activation of apoptosis-related pathways induced by nanoemulsions of cannabinoids. It is important to stress that immunohistochemical detection of cleaved caspase-3 reflects increased expression of an apoptotic marker [27]. Previous studies have demonstrated that cannabinoids may modulate apoptotic and proliferative pathways in a tissue-dependent manner, although the underlying mechanisms remain incompletely understood. For example, THC exposure has been associated with modulation of caspase-3 expression in reproductive cells [28], while other studies have reported variable effects of cannabinoid compounds on cell proliferation depending on cell type and experimental context [29,30]. In this context, the present findings suggest that subchronic exposure to higher doses of CBD and CBD+THC nanoemulsions may influence renal cellular homeostasis, although causal mechanisms cannot be established based on the current experimental design.

GST-P expression was significantly increased in the CBD 5 mg/kg group, suggesting activation of cellular stress or detoxification-related responses. Although GST-P is classically used as a marker in hepatic and preneoplastic models, it has also been applied in experimental toxicology as an indicator of cellular stress responses. In the present study, its increased expression may reflect adaptive responses to xenobiotic exposure [23]; however, in the absence of complementary molecular or functional data, no conclusions can be drawn regarding preneoplastic transformation or long-term pathological progression.

It is important to highlight that the present study evaluated morphological and immunohistochemical endpoints only. No functional renal parameters (such as serum creatinine, urea levels, or urinary biomarkers), oxidative stress markers, or inflammatory cytokines were assessed. Therefore, although structural and molecular alterations were observed at higher doses, these findings are insufficient to establish renal dysfunction or definitive nephrotoxicity. Another relevant consideration is the use of nanoemulsion-based delivery systems, which may modify the pharmacokinetic and biodistribution profiles of cannabinoids. Although the vehicle control group did not show significant alterations, potential interactions between formulation components and cannabinoids cannot be fully excluded. Additionally, nanoemulsion systems may enhance systemic exposure, which could contribute to the observed dose-dependent effects [20,21].

A limitation of this study is the exclusive use of male animals, which may restrict extrapolation to females due to potential sex-related differences in cannabinoid metabolism and renal susceptibility. Furthermore, the absence of a THC-only comparison group limits the ability to fully disentangle individual contributions of each cannabinoid in combined formulations.

Finally, it is important to emphasize that the present study investigated isolated cannabinoids (CBD and Δ^9^-THC) delivered via nanoemulsion systems. Therefore, the findings cannot be directly extrapolated to full-spectrum cannabis extracts, which contain multiple bioactive compounds that may act synergistically through the so-called entourage effect, potentially resulting in different biological outcomes [3,4].

## 5. Conclusions

In conclusion, subchronic exposure to higher doses of CBD and CBD+THC nanoemulsions was associated with histopathological and immunohistochemical alterations in renal tissue of male Wistar rats under experimental conditions. No significant alterations were observed at lower doses. However, in the absence of functional renal assessments, these findings should be interpreted as indicators of tissue-level and cellular responses rather than definitive evidence of nephrotoxicity. Further studies incorporating functional, biochemical, and mechanistic approaches are required to fully elucidate the renal safety profile of cannabinoid-based nanoformulations.

## Figures and Tables

**Table 1:** Histological scores from rat kidneys treated with Cannabis nanoemulsions over 21 days.

Scores	0	1	2	3
**CTRL**	8	0	0	0
**CBD 2.5**	4	4	0	0
CBD 5 *	0	0	0	8
**CBD+THC 2.5**	0	8	0	0
CBD+THC 5 *	0	0	1	7

Groups showing statistically significant differences compared to the control group are indicated by (*), *p* < 0.05 (Kruskal–Wallis test followed by Dunn’s post hoc test).

**Table 2:** Scores from cleaved caspase 3 expression in kidney of rats treated with cannabis nanoemulsions over 21 days.

Scores	Nº of Animals	SCORE			
		0	1	2	3
**CTRL**	5	1	4	0	0
**CBD 2.5 mg/kg**	5	0	2	3	0
CBD 5 mg/kg *	5	0	1	1	3
**CBD+THC 2.5 mg/kg**	5	2	3	0	0
CBD+THC 5 mg/kg *	5	0	0	3	2

Groups showing statistically significant differences compared to the control group are indicated by (*), *p* < 0.05 (Kruskal–Wallis test followed by Dunn’s post hoc test).

## Data Availability

The original contributions presented in this study are included in the article. Further inquiries can be directed to the corresponding author.
